# Maximum Power Point Tracking of Photovoltaic Generation System Using Improved Quantum-Behavior Particle Swarm Optimization

**DOI:** 10.3390/biomimetics9040223

**Published:** 2024-04-08

**Authors:** Gwo-Ruey Yu, Yong-Dong Chang, Weng-Sheng Lee

**Affiliations:** 1 Department of Electrical Engineering, National Chung Cheng University, Chia-Yi 62102, Taiwan; 2 Advanced Institute of Manufacturing with High-Tech Innovations, National Chung Cheng University, Chia-Yi 62102, Taiwan; 3 Department of Electrical Engineering, National Penghu University of Science and Technology, Magong 88046, Taiwan; cc890j096@gmail.com

**Keywords:** photovoltaic generation systems, maximum power point tracking, improved quantum-behavior particle swarm optimization

## Abstract

This study introduces an improved quantum-behavior particle swarm optimization (IQPSO), tailored for the task of maximum power point tracking (MPPT) within photovoltaic generation systems (PGSs). The power stage of the MPPT system comprises a series of buck-boost converters, while the control stage contains a microprocessor executing the biomimetic algorithm. Leveraging the series buck-boost converter, the MPPT system achieves optimal operation at the maximum power point under both ideal ambient conditions and partial shade conditions (PSCs). The proposed IQPSO addresses the premature convergence issue of QPSO, enhancing tracking accuracy and reducing tracking time by estimating the maximum power point and adjusting the probability distribution. Employing exponential decay, IQPSO facilitates a reduction in tracking time, consequently enhancing convergence efficiency and search capability. Through single-peak experiments, multi-peak experiments, irradiance-changing experiments, and full-day experiments, it is demonstrated that the tracking accuracy and tracking time of IQPSO outperform existing biomimetic algorithms, such as the QPSO, firefly algorithm (FA), and PSO.

## 1. Introduction

In recent years, solar power has witnessed exponential growth attributed to its myriad advantages, including environmental friendliness, minimal operational costs, and maintenance-free characteristics [1]. Consequently, there has been a notable proliferation of photovoltaic (PV) power generation systems, driven by substantial investments and diverse financial incentives offered by numerous countries [2]. Therefore, numerous studies related to solar panel applications exist in the literature, such as the design of solar panel tracking systems aimed at tracking the path of the sun to maximize the collection of radiant heat energy [3,4]. However, the power–voltage (P–V) curve represents a nonlinear system, resulting in a maximum power point on the P–V curve [5]. It is essential to develop a maximum power point tracking (MPPT) system to operate the photovoltaic generation system (PGS) at this maximum power point (MPP). Additionally, environmental conditions can alter the P–V curve. The P–V curve exhibits nonlinearity with changes in environmental conditions, specifically in temperature and solar irradiance. In scenarios characterized by partial shade conditions (PSCs), the P–V curve may exhibit multiple peaks, consequently heightening the intricacy associated with the MPPT technique [6,7]. Accordingly, operating the MPPT at MPP under changing environmental conditions and PSC is a focal point of the MPPT method.

To date, several MPPT methodologies have been proposed, such as the perturb and observe (P and O) technique [8,9,10], the hill climbing approach [11,12], and the incremental conductance method [13,14], among others. Despite their simple calculations, these methods may fail to accurately reach the MPP and achieve a balance between tracking speed and steady-state oscillation. Some researchers have applied fuzzy control to MPPT [15,16,17], but this requires establishing an MPPT model and time-consuming modifications to the control system. Additionally, constructing the PSC module using fuzzy modeling presents challenges. Neural networks have the capability to model complex dynamic systems, such as the inverse kinematics of a robotic arm [18]. Other researchers have employed neural networks for MPP tracking [19,20], but this method consumes significant memory and time to track the MPP. On the other hand, biomimetic algorithms are a robust tool for global optimization, suitable for complex problems, as they do not require extensive analysis like Monte Carlo methods [21]. In recent years, a plethora of bio-inspired metaheuristic algorithms have been proposed for addressing optimization problems. For instance, the Liver Cancer Algorithm employs an evolutionary search methodology that simulates the growth and progression dynamics of liver tumors, showcasing commendable efficacy in feature selection [22]. The Slime Mold Algorithm, derived from the natural oscillation patterns of slime molds, has developed into a stochastic optimizer renowned for its exceptional exploration abilities and exploitation tendencies [23]. The Moth Search Algorithm explores engineering optimization and applications based on the most representative features of moths, such as moth flight and phototaxis [24]. The Colony Predation Algorithm formulates stochastic optimization strategies by emulating the hunting tactics employed by animal groups, wherein boundary values are substituted with optimal position values across boundaries to augment the algorithm’s exploitation prowess [25]. In reference [26], the INFO Algorithm is introduced and implemented for global optimization, enhancing the updating rule and vector combination through the utilization of an improved weighted mean methodology. In reference [27], inspired by the cooperative behavior and chasing styles of Harris’s hawks in nature, the Harris Hawks Optimizer is proposed and applied to several engineering design problems. In reference [28], by simulating the growth and crossover behavior of rime-rice swarms, the RIME optimization algorithm is proposed, demonstrating good convergence accuracy and speed.

Some researchers have treated MPPT as an optimization problem and controlled it using particle swarm optimization (PSO) [29] or other biomimetic algorithms [30,31,32]. Compared to other MPPT methods, biomimetic algorithms are suitable for long-term operation in MPPT systems. They can mitigate feedback ratio distortion, thereby improving tracking accuracy. Among the array of bio-inspired algorithms, PSO emerges as notably well-suited for implementation in MPPT within PGS. The PSO algorithm offers several advantages, including simple calculations, low memory requirements, and easy programming, making it more promising for MPPT systems. PSO is a relatively simple algorithm, featuring ease of implementation and minimal hardware demands. This characteristic facilitates its deployment on ordinary microcontrollers or other low-cost platforms, obviating the necessity for high-end expensive chips. Such attributes bear considerable significance within the realm of practical photovoltaic generation engineering, offering substantial potential for cost reduction. Moreover, PSO commonly demonstrates accelerated computational speeds, enabling real-time modulation of output voltage or current to effectively trace the MPP of photovoltaic cells. This capability assumes critical importance in the context of PGS given the propensity for the MPP to fluctuate in response to external environmental conditions. Owing to its ability for online computation devoid of reliance on high-end expensive hardware, PSO emerges as a favored choice for conducting MPPT investigations within the domain of PGS. The traditional PSO algorithm exhibits steady-state oscillation, prolonging the tracking time and decreasing tracking accuracy. An improved PSO was proposed in the literature [33], which decayed the argument based on generation but still exhibited steady-state oscillation. In the study cited as reference [34], a modified PSO was employed within a MPPT system. Upon discovering a new global maximum, both the velocity and particle position were re-initialized. Nevertheless, the rationale behind the selection of criteria for resetting the arguments was not elucidated. In reference [35], a two-stage algorithm was proposed. In the first step, MPPT operated to the first peak using P and O, and in the second stage, MPPT searched the remaining space using P and O, but this method is only effective in multi-peak situations. In reference [36], another two-stage algorithm was proposed to reduce steady-state oscillation by reducing velocity when the particle approaches the MPP in PSO. When the velocity is less than the setup point, MPPT operates at MPP using P and O. This reduces steady-state oscillation, but it requires limiting the maximum velocity of particles and may still get stuck in local maximums in some situations. In reference [37], MPPT is operated using FA. Although FA has high-speed convergence, it must balance tracking time and accuracy.

The PSO algorithm still faces some challenges that need to be addressed, such as premature convergence and the trade-off between accuracy and efficiency. To enhance PSO, the quantum-behaved particle swarm optimization (QPSO) algorithm was introduced. It aims to meliorate global exploration by integrating principles from quantum mechanics into the PSO model [38]. QPSO utilizes quantum behavior instead of particle position and velocity in PSO. QPSO ensures that particles can occupy any position, thereby guaranteeing a good balance between tracking time and tracking accuracy. In the realm of global optimization problems, the QPSO algorithm demonstrates superior convergence performance in comparison to the PSO algorithm. Nonetheless, there persist several challenges associated with QPSO. Over time, numerous researchers have endeavored to enhance and implement QPSO in various domains. For instance, a pioneering approach involving a novel particle dimension search strategy has been proposed, aiming to transform the original evaluation function into a path evaluation point function for route planning of fixed-wing UAV [39]. Furthermore, in the literature [40], an improved version of the QPSO algorithm has been employed to train a wavelet neural network for anomaly detection in large-scale multimedia data transmission networks. Additionally, reference [41] introduces an enhanced QPSO algorithm incorporating a large-to-small contraction-expansion coefficient strategy, specifically tailored to tackle the inverse kinematics problem encountered in robotics. Furthermore, this study devises a control allocation scheme utilizing the improved QPSO algorithm to calculate both the thrust and rotation angle of the manned submersible, thereby contributing to the effective minimization of energy consumption within the thruster system [42]. However, it is worth noting that despite these advancements, challenges such as the occurrence of local optima and sluggish convergence persist, particularly as the scale of the problem enlarges.

This study presents the design of an improved quantum-behavior particle swarm optimization (IQPSO) algorithm aimed at mitigating the premature convergence issue inherent in traditional QPSO approaches, while simultaneously augmenting the convergence speed and tracking accuracy of QPSO. To expedite convergence, a natural exponential decay method is employed for the contraction–expansion coefficient, while accuracy enhancements are achieved through the estimation of probability distributions and optimal solution positions that evolve with each generation. By integrating biomimetic algorithms with a series buck-boost converter, the system can operate in both buck and boost modes. The IQPSO algorithm is then implemented into a chip for application in MPPT systems, facilitating PGS to operate at their maximum power point despite varying irradiance and shading conditions. Subsequently, MPPT experiments are conducted using a DC programmable power supply to simulate solar photovoltaic systems, with comparisons made against other biomimetic algorithms such as the QPSO, FA, and PSO. Through single-peak experiments, multi-peak experiments, and irradiance variation experiments, the superiority of IQPSO in tracking accuracy and tracking speed is verified. Finally, practical testing involving single-peak, multi-peak, and full-day assessments of PV arrays validate the superior performance of IQPSO over other biomimetic algorithms under real-world conditions.

The principal contributions of this article are outlined as follows. Primarily, the introduction of IQPSO addresses the challenge of premature convergence observed in traditional QPSO methodologies, thereby enhancing both convergence speed and tracking accuracy. Secondly, IQPSO demonstrates superior MPPT performance in scenarios involving single-peak, multi-peak, and changing irradiance. Lastly, the efficacy of IQPSO in practical PGS is substantiated through comprehensive full-day testing, highlighting its ability to enhance the power generation efficiency of PGS under realistic operating conditions.

## 2. The PV Circuit and the Effects of Environmental Conditions

### 2.1. The Equivalent Circuit of PV Cells

**Figure 1:**
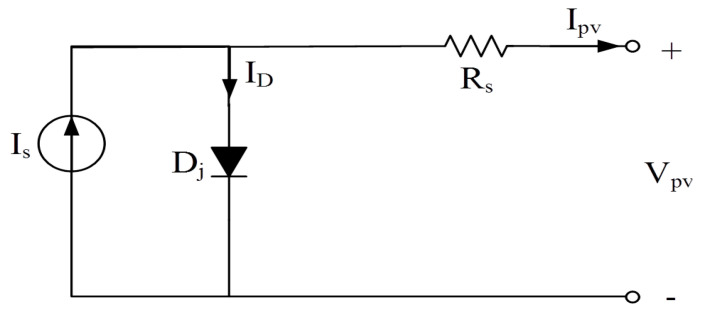
Equivalent circuit of PV cells.

**Figure 2:**
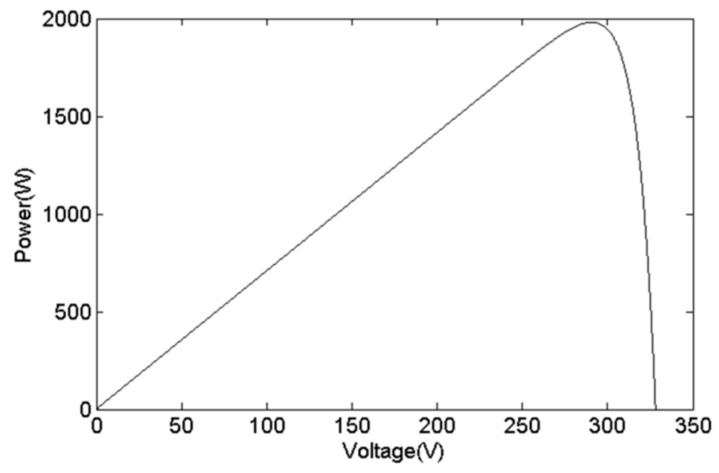
Power–Voltage curve of PV cells.

PV cells are composed of multiple P–N junction semiconductors. Illustrated in Figure 1 is an equivalent circuit representing PV cells. This model includes a current source *I_s_*, a parallel diode *D_j_*, a series resistance *R_s_*, a *PV* output current *I_pv_*, and a PV output voltage *V_pv_*. The output current can be represented by Equation (1), where *k* is the Boltzmann’s constant, *n* is the ideality factor, *T* is the temperature in Kelvin, *q* is the electron charge, and *I_d_* is the saturation current. Thus, the output power of the PV cells can be obtained as Equation (2). Figure 2 shows the Power–Voltage (P–V) curve of PV cells, which is a nonlinear curve due to the MPP.

(1) $$I_{pv}=I_{s}-I_{d}\left(\exp\left(\frac{q\left(V_{pv}+I_{pv}\times R_{s}\right)}{nkT}\right)-1\right)$$

(2) $$P_{pv}=I_{pv}V_{pv}=I_{s}V_{pv}-I_{d}V_{pv}\left(\exp\left(\frac{q\left(V_{pv}+I_{pv}\times R_{s}\right)}{nkT}\right)-1\right)$$

### 2.2. The Effects of Irradiance, Temperature, and PSC

**Figure 3:**
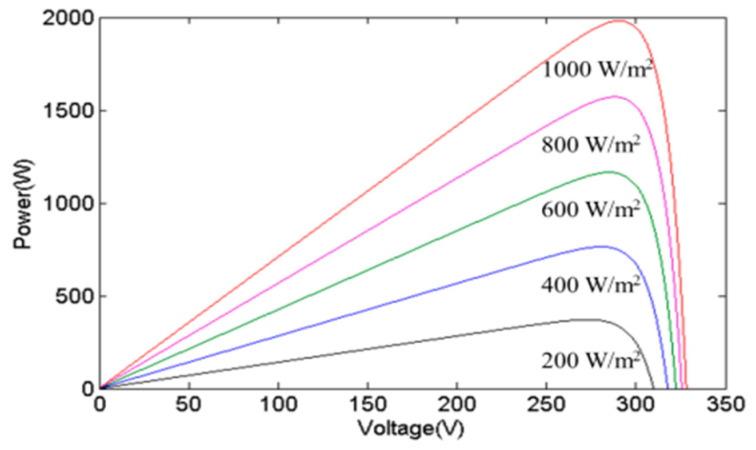
The effects of various irradiances.

**Figure 4:**
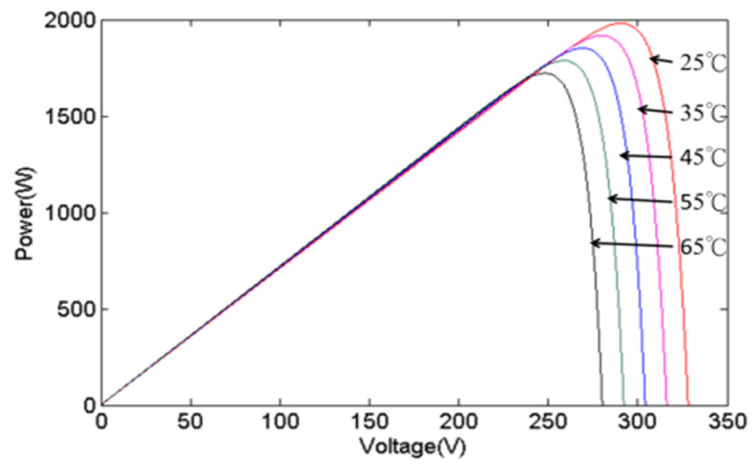
The effects of various temperatures.

The inherent limitations of individual photovoltaic (PV) cells, such as their low output voltage and current, render them impractical for direct application. Consequently, PGSs typically consist of several PV cells to raise the output current and voltage. The power curve of a PGS will vary due to changes in irradiance and temperature. Figure 3 and Figure 4, respectively, show the changes in the power curve of a PGS when affected by irradiance and temperature. When irradiance changes, the maximum power sharply decreases as irradiance decreases, while the MPP voltage only shifts slightly to the left and decreases. When the temperature changes, the maximum power decreases as temperature increases, and the MPP voltage shifts to the left and becomes smaller. These observations underscore the significant influence of irradiance on the output power of a PGS, as elucidated by the depicted data in Figure 3 and Figure 4.

**Figure 5:**
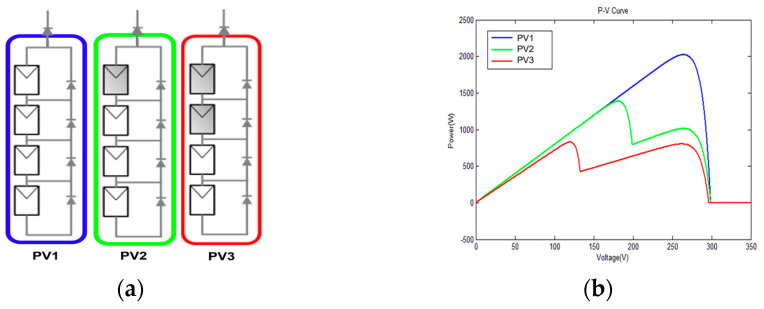
The effects of PSC: (**a**) PV panels; (**b**) power curves.

In a PGS, if some PV cells are shaded, resulting in the decreased power generation of certain PV panels, connecting them in series and parallel with unshaded PV panels can lead to multi-peak conditions in the overall power curve. Figure 5a illustrates a schematic diagram of the photovoltaic panels under different shading conditions. Blue indicates no shading, green indicates a small amount of shading, and red indicates significant shading. Subsequently, Figure 5b presents the resultant power curve derived from the configurations outlined in Figure 5a. When the PV panels are completely unshaded, the power curve of the PGS exhibits a single blue peak. Conversely, when a small part of the PV panels is shaded, the power curve of the PGS shows a green double peak. In scenarios where shading predominantly impacts most of the PV panels, the resulting power curve assumes a red multi-peaked characteristic. Based on the above, effectively operating the PGS at the maximum power point under different conditions due to irradiance, temperature changes, and partial shading poses a challenge for the MPPT approach in terms of both speed and accuracy.

## 3. IQPSO Algorithm

### 3.1. QPSO Algorithm

The QPSO algorithm integrates principles derived from quantum mechanics and utilizes quantum behavior to describe the velocity and position of particles within the PSO optimization framework. Equations (3) to (7) represent the formulas of the traditional QPSO algorithm [31]. In Equation (3), $x_{i}^{k+1}$ denotes the new position of the *i*th particle at the next iteration, $P_{i}^{k}$ represents the local attractor at the present iteration, $B_{i}^{k}$ denotes the characteristic length of the delta potential well at the present iteration, and *k* indicates the current iteration number. In Equation (4), β denotes the contraction–expansion coefficient. In Equation (5), $p_{best,i}$ denotes the best position of the *i*th particle, $g_{best}$ denotes the best position of the particle swarm, and *r_1_* is a random variable in the interval [0, 1]. In Equation (6), $M^{k}$ denotes the mean best position of the particle swarm at the present iteration, and *u* is a random variable within [0, 1]. The value of the random variable *u* determines the sign in the Equation (3). If *u* is greater than 0.5, a negative sign is adopted; otherwise, a positive sign is used in Equation (3). In Equation (7), *N* denotes the number of particles. The QPSO algorithm fundamentally operates based on the quantum motion of the delta potential well. The Monte Carlo approach is integrated to guarantee a balanced exploration–exploitation trade-off during the search process. The particles have a certain probability of being dispersed throughout the search area, increasing the likelihood of finding the globally optimal solution across the entire domain.

(3) $$x_{i}^{k+1}=P_{i}^{k}\pm B_{i}^{k}$$

(4) $$\beta =\beta_{max}-\frac{k}{k_{max}}\times (\beta_{max}-\beta_{min})$$

(5) $$P_{i}^{k}=r_{1}p_{best,i}+(1-r_{1})g_{best}$$

(6) $$B_{i}^{k}=\beta \times \left|M^{k}-x_{i}^{k}\right|\times \ln\left(\frac{1}{u}\right)$$

(7) $$M^{k}=\sum_{i=1}^{N}\frac{p_{best,i}}{N}$$

### 3.2. IQPSO Algorithm

This paper proposes the IQPSO algorithm to address the premature convergence issue of traditional QPSO from four perspectives, namely, the contraction–expansion coefficient *β*, the range of random variables for local attractors $P_{i}^{k}$, the characteristic length $B_{i}^{k}$ of the delta potential well, and the mean best position *M*^k^ of the particle swarm. To expedite the convergence speed of the QPSO algorithm, the contraction–expansion coefficient *β* is typically designed to decrease with the increase in generations, as depicted in Equation (4). However, during the initial phases of the QPSO algorithm, *β* may easily lead particles to exceed the search space and become confined to the search boundary. In the later stages of the QPSO algorithm, if *β* becomes too small when nearing the steady state, it may impede particle mobility, causing them to linger near the optimal point and failing to attain the true optimal value. Consequently, this may result in premature convergence issues, leading to the failure to attain the globally optimal solution. To circumvent these premature convergence problems, this study exploits the characteristic that the reciprocal of the natural exponent with a small power has a larger value in the early stages of the algorithm and gradually decreases to a positive number that is significant as it approaches the steady state. Equation (4) is refined into to Equation (8), enabling the contraction–expansion coefficient *β* to decrease in a natural exponential fashion as the number of iterations increases. Consequently, the proposed improved QPSO algorithm can expedite the convergence speed in the early stages and enhance tracking accuracy in the steady state.

(8) $$\beta =\exp\left(-\gamma \frac{k}{k_{max}}\right)$$

where *γ* is a preset positive number.

**Figure 6:**
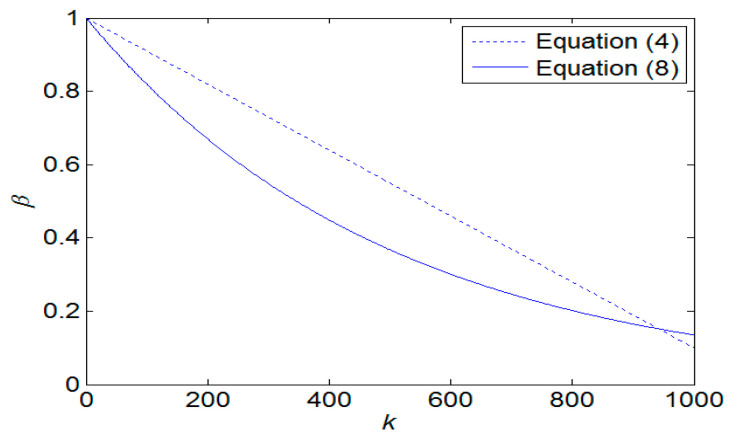
The comparison of *β* values using Equations (4) and (8).

**Figure 7:**
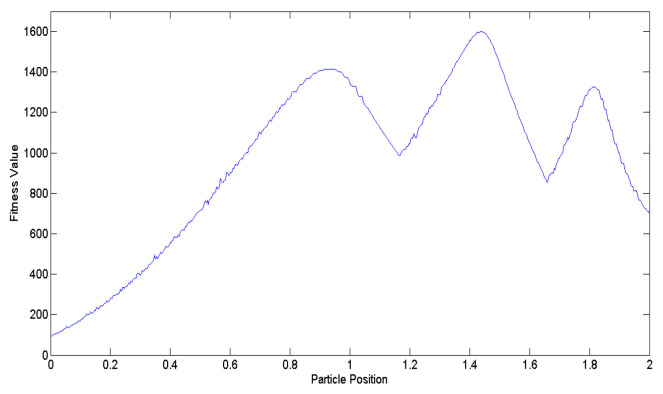
The multi-peak objective function.

**Figure 8:**
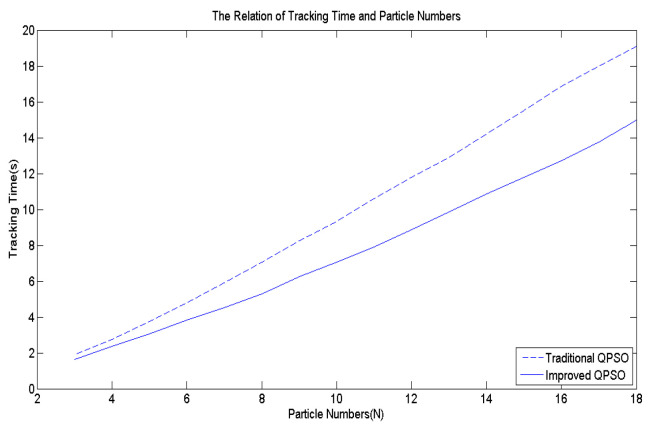
The effects of exponential *β*.

Figure 6 illustrates a comparative analysis of *β* values obtained using Equations (4) and (8). During the transient state, the *β* values derived from Equation (8) are smaller than those derived from Equation (4). However, as the number of iterations approaches the steady state, the *β* values of Equation (4) will become significantly smaller because Equation (4) decreases in a linear manner. In contrast, Equation (8) exhibits exponential decrease, ensuring that the *β* value does not become too small. To validate the effectiveness of Equation (8), a multi-peak function shown in Figure 7 is employed as the objective function for comparison. Following 10,000 simulation runs, the average tracking time was calculated, as shown in Figure 8. It is evident that regardless of the number of particles utilized, the average tracking time associated with the modified contraction–expansion coefficient proposed in this article is notably shorter compared to that associated with the traditional contraction–expansion coefficient.

Moreover, the significance of the optimal solution of the particle swarm increases with the progression of iterations. In the current QPSO algorithm, Equation (5) solely employs random variables to strike a balance between the optimal solution of individual particles and that of the particle swarm, without adjusting its weight with the iteration count. Consequently, this approach may predispose the algorithm towards convergence to a local optimal solution, thus failing to achieve the global optimal solution. To rectify this limitation, this paper leverages the characteristic of the decreasing *β* parameter as the number of iterations increases. Equation (5) is amended to Equation (9), wherein the range of the random variable $r_{1}$ is adjusted to [0, *β*] to enhance the weighting of the optimal solution of the particle swarm in the later stages of the algorithm.

(9) $$P_{i}^{k}=r_{1}p_{best,i}+(1-r_{1})g_{best}\;r_{1}\in \left[\begin{array}{cc} 0 & \beta \end{array}\right]$$

where $r_{1}$ is a random variable.

**Figure 9:**
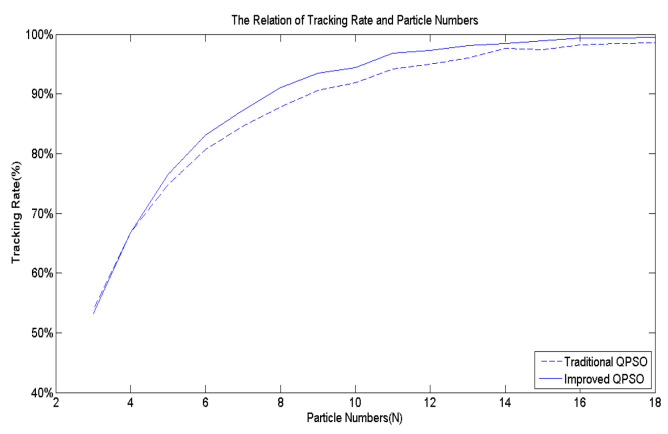
The effects of *r*_1_.

Figure 7 serves as the benchmark multi-peak objective function utilized to assess the effectiveness of Equation (9). Following 10,000 simulation runs, the average success rate of tracking the best solution across the entire domain is computed. Figure 9 illustrates that regardless of the number of particles, the average success rate achieved by Equation (9) proposed in this study surpasses that of the existing Equation (5).

During the initial phase of the conventional QPSO algorithm, while Equation (6) aids particles in escaping from locally optimal solutions, it also elevates the likelihood of particles not converging easily in the later stages of the algorithm. This phenomenon may result in increased tracking time during the application of MPPT, thereby resulting in a power loss. To mitigate this issue, this paper capitalizes on the characteristic of *β* decreasing with generations and transforms Equation (6) into Equation (10), wherein $B_{i}^{k}$ is gradually reduced in the later stages of the algorithm to hasten the convergence speed of particles.

(10) $$B_{i}^{k}=\beta \times \left|M^{k}-x_{i}^{k}\right|\times \ln\left(\frac{1}{1-u\times \beta }\right)$$

**Figure 10:**
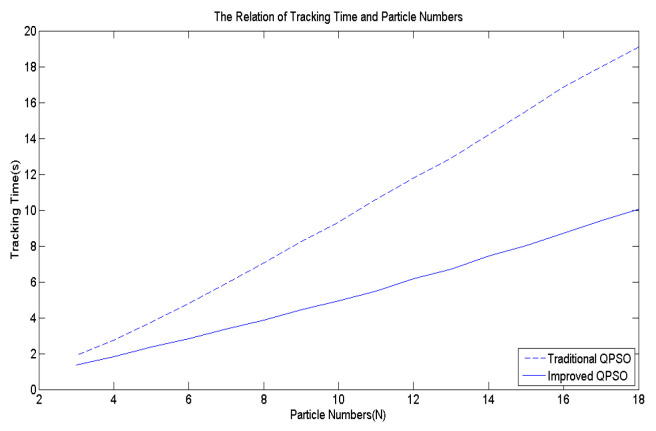
The effects of $B_{i}^{k}$.

To ascertain the efficacy of Equation (10), Figure 7 is employed as the multi-peak objective function to compute the average convergence speed after 10,000 simulation runs. Figure 10 depicts the comparison of the average convergence time between the proposed method and the traditional one. The findings unequivocally reveal that irrespective of the number of particles, the approach proposed in this paper achieves much faster convergence than the existing method.

Additionally, within the traditional QPSO algorithm, *M^k^* represents, solely, the mean’s best position of the particle swarm, as delineated in Equation (7). However, this calculation of *M^k^* does not consider the relationship between the fitness value and the movement of individual particles towards the best position of the particle swarm. Furthermore, if the particle positions are unevenly distributed, the mean best value of the particle swarm may be distant from the globally optimal solution, thereby resulting in slower convergence or even convergence only to a locally optimal solution. Consequently, this paper comprehensively considers the fitness value, individual particle position, and the best position of the particle swarm to modify *M^k^*, as depicted in Equation (11), aiming to enhance the success rate of tracking within the proposed QPSO algorithm.

(11) $$M^{k}=g_{best}+\frac{\sum_{i=1}^{N}\left[\left(F_{i}-F_{gbest}\right)\times \left(x_{i}^{k}-g_{best}\right)\right]}{\left|\sum_{i=1}^{N}\left(F_{i}-F_{gbest}\right)\right|}$$

**Figure 11:**
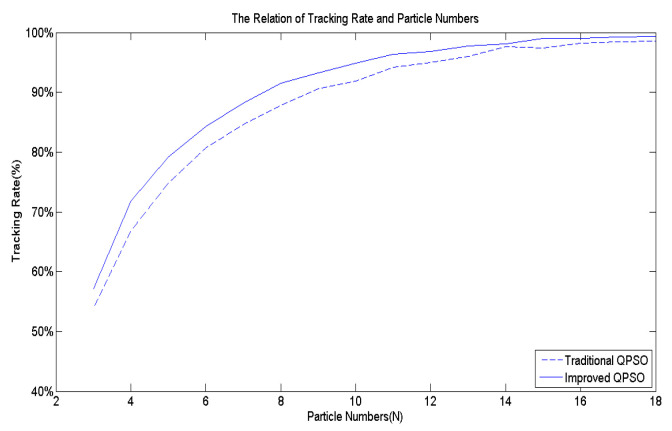
The effects of *M^k^*.

Figure 7 shows the multi-peak objective function utilized to validate the efficacy of Equation (11). Subsequently, Figure 11 illustrates the average tracking rate of the search for the globally optimal solution after 10,000 simulation runs. The proposed *M^k^* exhibits a superior tracking rate compared to the existing *M^k^*, regardless of the number of particles utilized in the algorithm.

**Figure 12:**
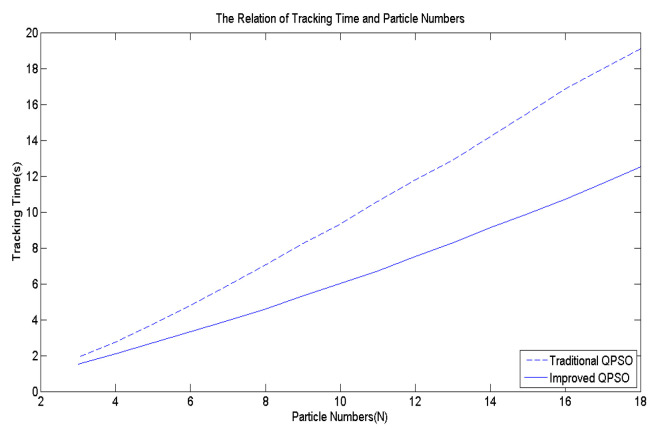
The tracking time comparisons of IQPSO and QPSO.

**Figure 13:**
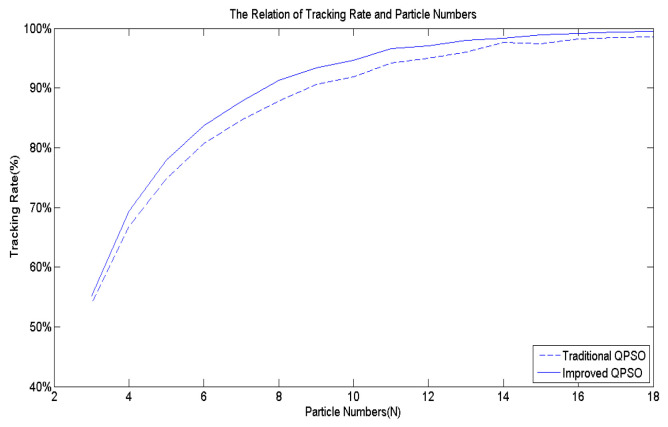
The tracking rate comparisons of IQPSO and QPSO.

Building upon the insights, this paper devises an improved QPSO algorithm, incorporating Equations (3) along with Equations (8) to (11), thereby enhancing the tracking accuracy and speed of the proposed biomimetic method. Figure 7 serves as the benchmark multi-peak objective function to contrast the IQPSO algorithm with the QPSO algorithm, with γ set to 20. Through 10,000 simulation iterations, the tracking time and success rate are illustrated in Figure 12 and Figure 13, respectively. The findings conclude that the proposed IQPSO algorithm surpasses the existing QPSO algorithm and is better suited for intricate search and tracking tasks.

**Figure 14:**
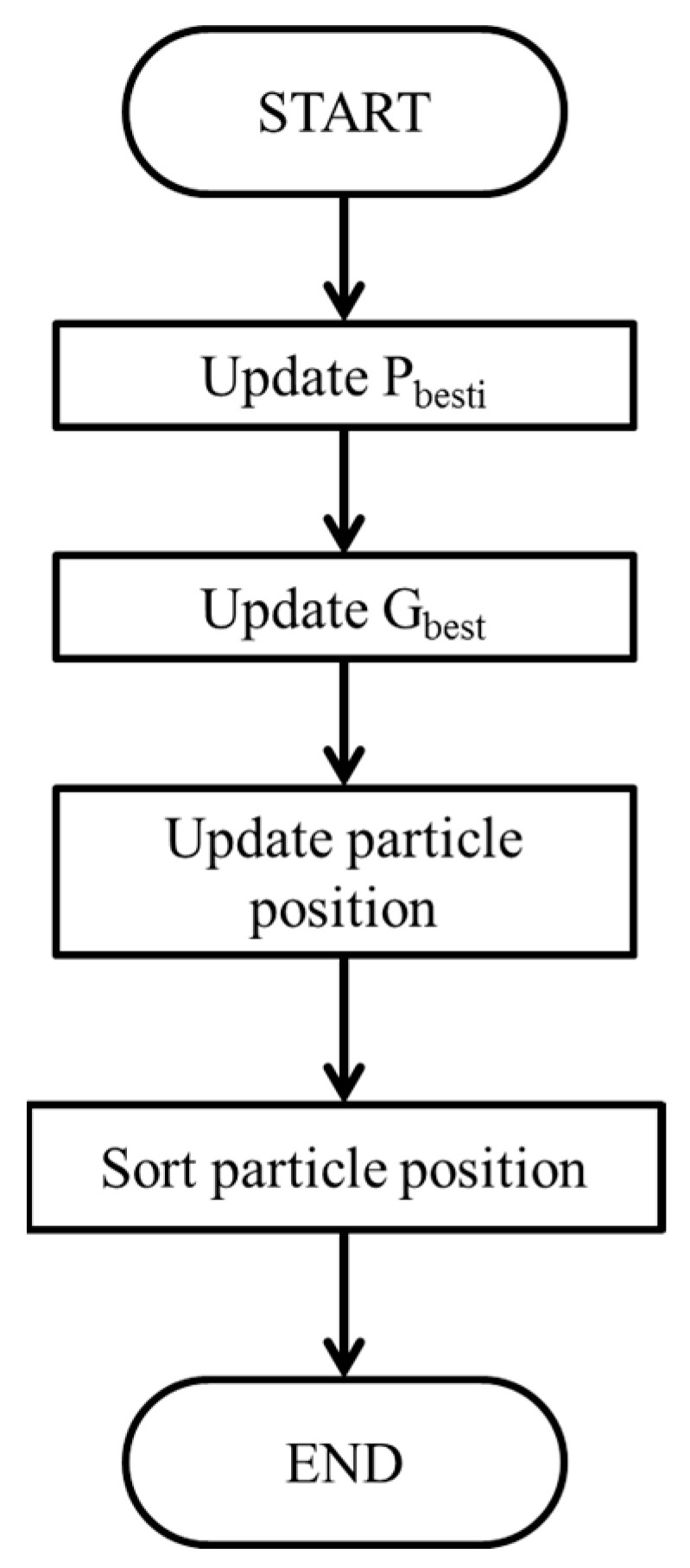
Flowchart of IQPSO.

Figure 14 shows the flowchart depicting the proposed IQPSO. The optimization process and description of IQPSO are presented herein. The process is outlined as follows:

*Step 1 IQPSO Initialization*: The IQPSO initialization involves setting parameters such as the particle number *N* and the convergence coefficient *γ*. The larger the particle number *N*, the longer the tracking time and the higher the tracking rate. The *γ* must be preset according to various system characteristics. Typically, particles are initialized with a random distribution over the search space.

*Step 2 Fitness Evaluation*: Calculate the fitness value of each particle. The fitness function is defined as the power of the PV array.

*Step 3 Update* $p_{best,i}$ *and* $g_{best}$: Based on the fitness value obtained in *Step 2*, the best position of the *i*th particle ($p_{best,i}$) and the best position of the particle swarm ($g_{best}$) are updated, respectively.

*Step 4 Update Particle Position*: Utilizing Equation (3), the particle positions are updated incorporating Equations (8) to (11).

*Step 5 Sort Particle Position*: The particle positions are sorted to speed up the convergence of maximum power tracking.

## 4. MPPT Circuit

**Figure 15:**
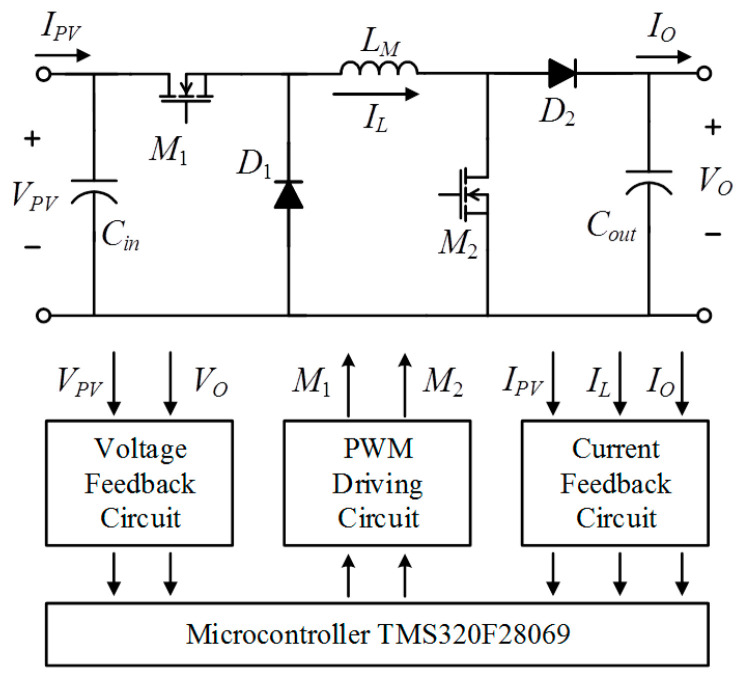
The topology of the MPPT circuit.

This paper employs a series buck-boost converter for MPPT in a PGS. Figure 15 shows the circuit diagram of the series buck-boost converter along with its associated peripheral circuits. Table 1 presents an overview of the specifications of the MPPT circuit. The series buck-boost converter comprises a buck converter and a booster converter, regulating the power switch *M*_1_ into buck mode and the power switch *M*_2_ into boost mode, respectively. Upon receiving the signals *Vpv* and *Ipv* from the feedback circuits, the microprocessor TMS320F28069 calculates the duty ratio using the IQPSO program stored within the chip. Based on the calculated duty ratio, the corresponding pulse width modulation (PWM) signal drives the insulated gate bipolar transistor (IGBT) power switch in either buck mode or boost mode, maximizing the output power of the PGS. 

**Figure 16:**
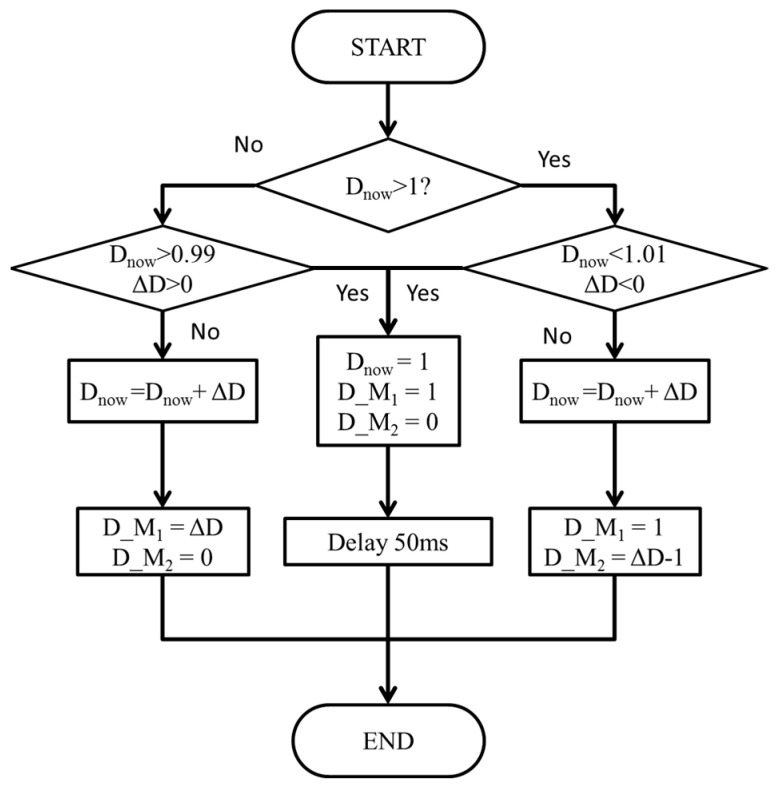
Flowchart of MPPT mode change.

To search for the MPP under varying irradiance and temperature conditions, the MPPT circuit must switch between buck and boost modes. In this paper, the duty ratio of the power switch is designed as the particle position, distributed within the range of [0, 2]. Specifically, the particle position [0, 1] indicates that the MPPT is operated in buck mode, wherein the duty ratio ranges from 0% to 100%. Conversely, the particle position [1, 2] signifies that the MPPT is operated in boost mode, with the duty ratio ranging from 0% to 100%. Figure 16 depicts the flowchart illustrating the mode conversion process. The symbol D_now_ represents the current particle position of MPPT, while ΔD represents the change step of the duty ratio. Additionally, the symbol D_M_1_ denotes the duty ratio of the power switch *M*_1_, and D_M_2_ denotes the duty ratio of the power switch *M_2_*. Throughout the MPPT mode switching process, D_M_1_ is set to 1, D_M_2_ is set to 0, and a delay of 50 ms is implemented to stabilize the input voltage before transitioning modes to reduce surges.

## 5. IQPSO MPPT

**Figure 17:**
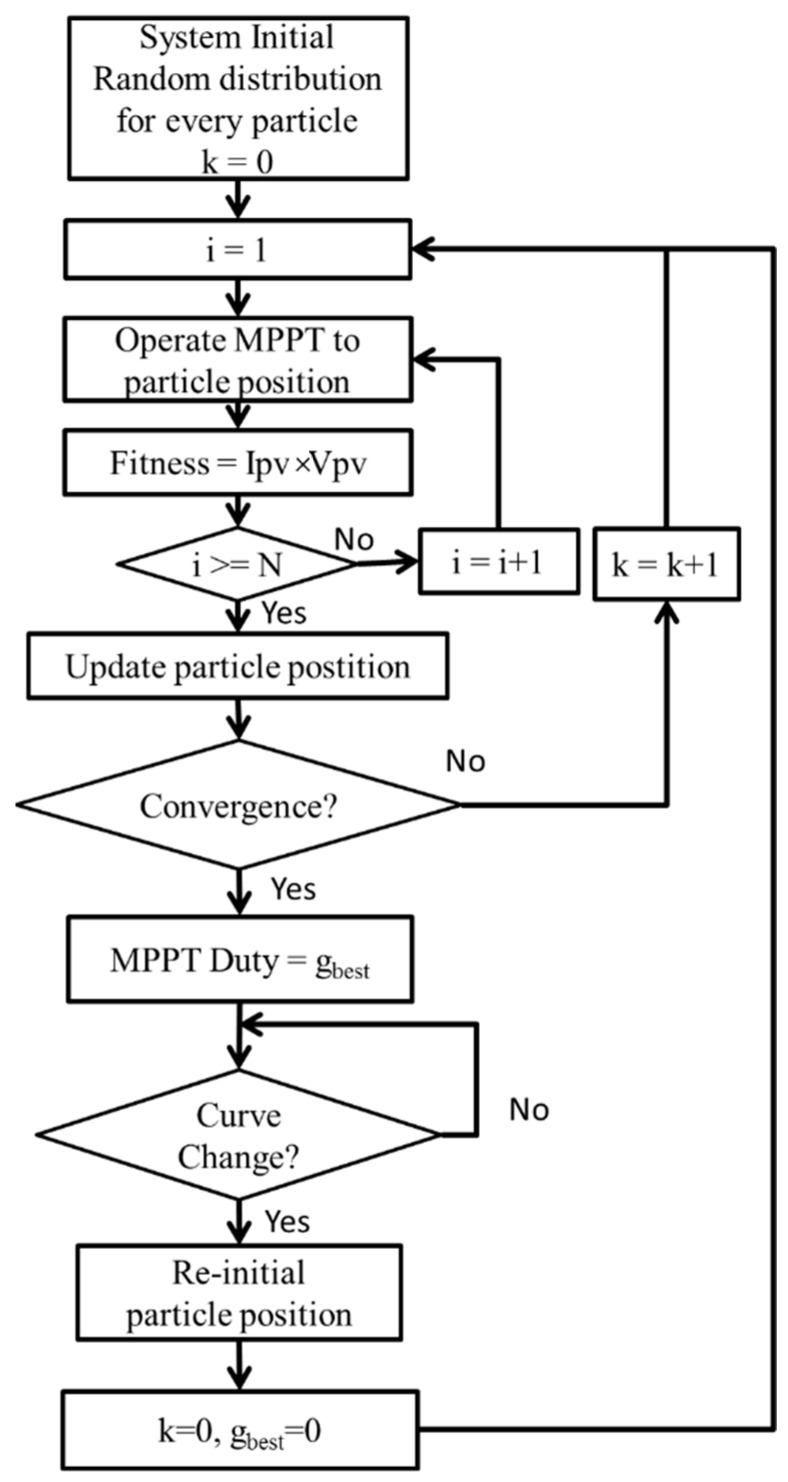
Flowchart of IQPSO MPPT.

Figure 17 shows the flowchart illustrating the IQPSO MPPT process. The procedure is outlined as follows:

*Step 1 System Initialization*: Commence by initializing the parameters and particle positions of the IQPSO algorithm, where *i* denotes the particle number and *k* denotes the generation number. Set the particle position through random distribution.

*Step 2 Fitness Calculation*: Operate the MPPT based on the particle positions and calculate the PV power as the fitness value.

*Step 3 IQPSO Execution*: Update the particle positions using the proposed IQPSO algorithm.

*Step 4 Convergence Judgment*: Determine whether the convergence condition has been met. In this study, the convergence condition is defined as the difference between the maximum particle position and the minimum particle position being less than 0.01. If the particle distribution falls below 0.01, it means that the IQPSO algorithm has converged, and the PGS will operate at the MPP. If convergence has not been achieved, return to step 2 for the next iteration.

*Step 5 Curve Change Judgment*: When the power curve fluctuates due to changes in irradiance, temperature, or partial shading, the PGS might not reach the maximum power output while operating at the initially identified global best particle position. This study defines a power curve change as the difference between the maximum power and the ideal value exceeding 1%. If the fitness variation surpasses 1%, indicating that the P–V curve requires modification, revert to step 1 for further exploration.

## 6. Experimental Results

**Figure 18:**
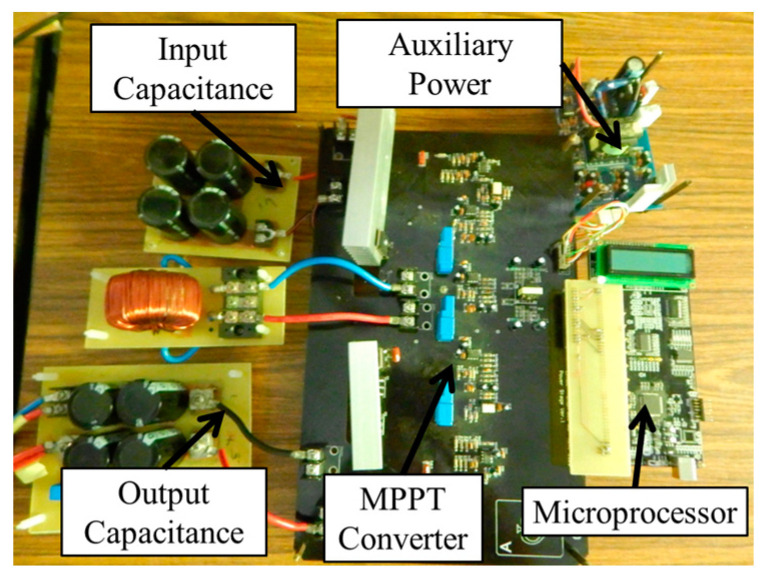
Hardware of the MPPT circuit system.

Figure 18 shows the hardware implementation of the MPPT circuit system. The 62100H-600S programmable DC power supply is utilized to simulate the solar photovoltaic system, facilitating the conduct of single-peak experiments, multi-peak experiments, and irradiance change experiments initially. Alongside the proposed IQPSO, the existing QPSO, FA, and PSO algorithms are also programmed into the microprocessor to enable the comparative analysis of MPPT performance. To ensure fairness in comparing the tracking results of various MPPT algorithms and considering computational time, the particle number is consistently set to 5. Table 2 outlines the parameter settings for the various MPPT algorithms. Given the stochastic nature of the four biomimetic algorithms, the experiments are repeated one thousand times to ascertain optimal parameters. Tracking time is defined as the duration required for the steady-state error to decrease to 1%, while tracking accuracy is quantified according to Equation (12).

(12) $$\eta =\frac{P_{PV,\;MPPT}}{P_{PV,\;GMAX}}$$

where $P_{PV,\;MPPT}$ denotes the PV output power searched through the MPPT algorithm, and $P_{PV,\;GMAX}$ denotes the globally maximum PV output power.

**Figure 19:**
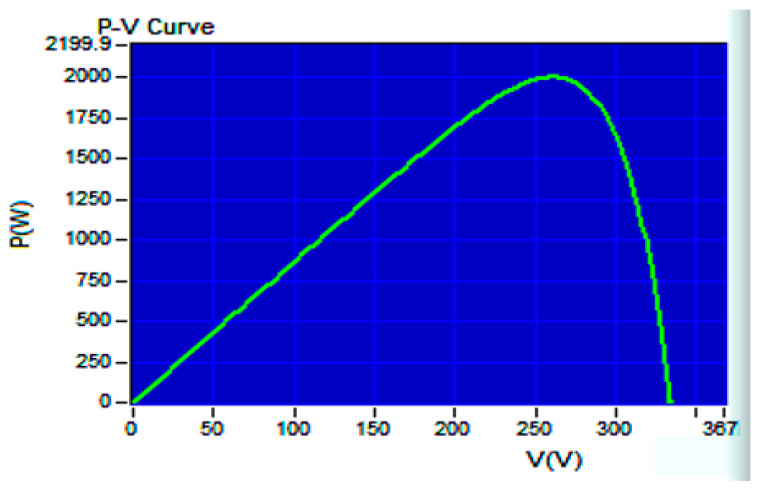
P–V curve in the case of a single-peak.

**Figure 20:**
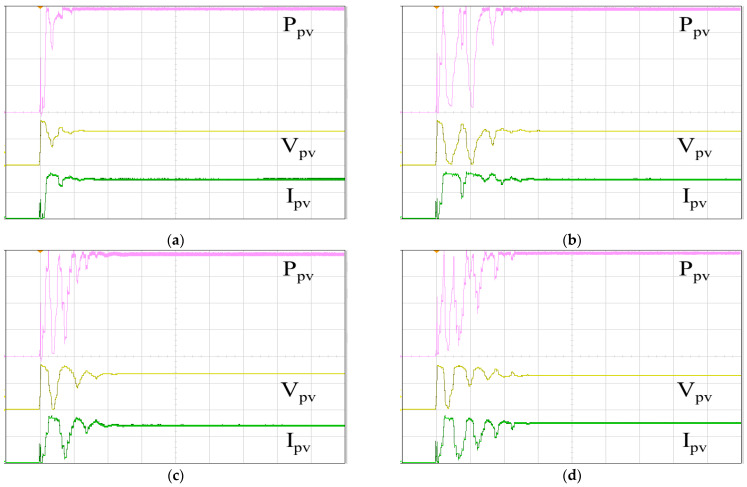
MPPT responses of the single-peak P–V curve: (**a**) IQPSO; (**b**) QPSO; (**c**) FA; (**d**) PSO (Ppv: 500 W/div, Vpv: 200 V/div, Ipv: 5 A/div, time: 2 s/div).

Figure 19 illustrates the P–V curve generated by the 62100H-600S programmable DC power supply, with an open circuit voltage of 333 V, a short-circuit current of 8.659 A, a MPP voltage of 260 V, and a MPP current of 7.692 A. Therefore, the global maximum PV output power $P_{PV,\;GMAX}$ is calculated to be 1999.92 W. In Figure 20, the tracking responses of the single-peak P–V curve using various MPPT algorithms are presented. Figure 20a displays the response of the IQPSO MPPT algorithm, which exhibits the fastest response and the highest accuracy among the algorithms considered. Figure 20b shows the response of the QPSO MPPT algorithm, which is slow but accurate. Figure 20c illustrates the response of the FA MPPT algorithm, characterized by its speed but lower accuracy. Figure 20d demonstrates the response of the PSO MPPT algorithm, featuring the slowest response and relatively lower accuracy. Table 3 summarizes the experimental results for the single-peak case for comparison. It is evident that the proposed IQPSO algorithm achieves the best MPPT performance in terms of both tracking accuracy and tracking time. Table 4 lists the ablation study for the single-peak case. It can be observed that the contraction–expansion coefficient has the most significant impact on the MPPT performance. A possible reason is that changes in *β* also induce modifications in Equations (9) and (10). Following this, the factor with a greater impact on MPPT performance is Equation (10), followed by Equation (11). Conversely, the factor with the smallest impact on MPPT performance is Equation (9), as the equation solely alters the range of the random variables.

**Figure 21:**
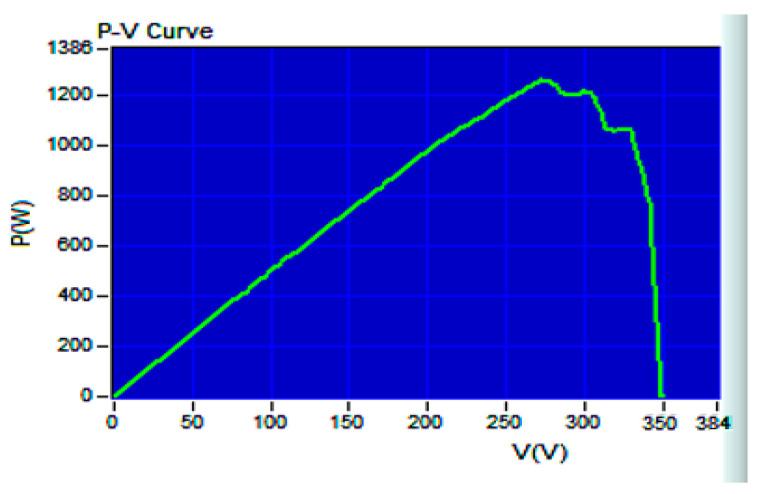
P–V curve in the case of a multi-peak.

**Figure 22:**
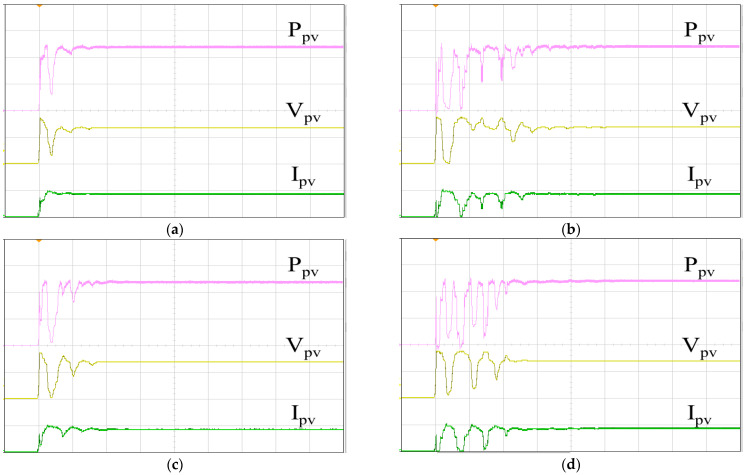
MPPT responses of the multi-peak P–V curve: (**a**) IQPSO; (**b**) QPSO; (**c**) FA; (**d**) PSO (Ppv: 500 W/div, Vpv: 200 V/div, Ipv: 5 A/div, time: 2 s/div).

Figure 21 shows the P–V curve in the case of a multi-peak, generated by the 62100H-600S programmable DC power supply, simulating a solar photovoltaic system under partial shading. The open circuit voltage is recorded as 349.5 V, with a short circuit current of 5.163 A, an MPP voltage of 272.39 V, and a MPP current of 4.6258 A. Therefore, the global maximum PV output power $P_{PV,\;GMAX}$ is calculated to be 1260.02 W. Figure 22 displays the tracking responses of the multi-peak P–V curve using various MPPT algorithms. Figure 22a exhibits the response of the IQPSO MPPT algorithm, which demonstrates the fastest response and the highest accuracy among the algorithms considered. Figure 22b shows the response of the QPSO MPPT algorithm, characterized by the slowest response but good accuracy. Figure 22c illustrates the response of the FA MPPT algorithm, which is fast but less accurate. Figure 22d demonstrates the response of the PSO MPPT algorithm, exhibiting the worst accuracy and is not very fast. Table 5 lists the experimental results in the multi-peak case for comparison. Clearly, in terms of both tracking accuracy and tracking time, the proposed IQPSO algorithm outperforms the others in the multi-peak scenario. Table 6 lists the results of the ablation study for the multi-peak case. It can be observed that the contraction–expansion coefficient still has the most significant impact on MPPT performance. Conversely, the mechanism with the least impact on MPPT performance remains Equation (9). Following this, the effects of other mechanisms on MPPT performance are observed in Equations (10) and (11) in sequence. These rankings align with those presented in Table 4.

**Figure 23:**
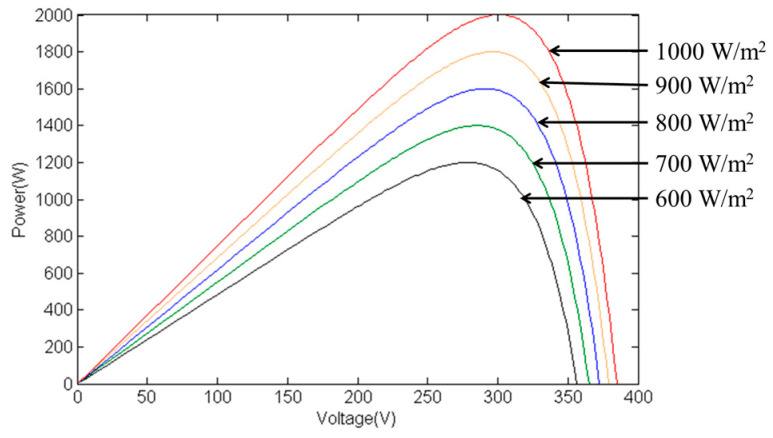
P–V curves under different irradiance conditions.

To assess the MPPT performance of the proposed IQPSO algorithm under changing irradiance conditions, this study implements an irradiance variation of 100 W/m^2^ every 10 seconds. Specifically, the irradiance decreases from 1000 W/m^2^ to 600 W/m^2^ and then increases back to 1000 W/m^2^ cyclically. The experiment lasts a total of ninety seconds. Experimental data obtained from the 62100H-600S programmable DC power supply is utilized to construct the power curve, as depicted in Figure 23. Throughout the irradiance decrease from 1000 W/m^2^ to 600 W/m^2^, the temperature remains fixed at 25 °C. The global maximum PV output power $P_{PV,\;GMAX}$ corresponds to 1999.92 W, 1800 W, 1600 W, 1400 W, and 1200 W, respectively.

**Figure 24:**
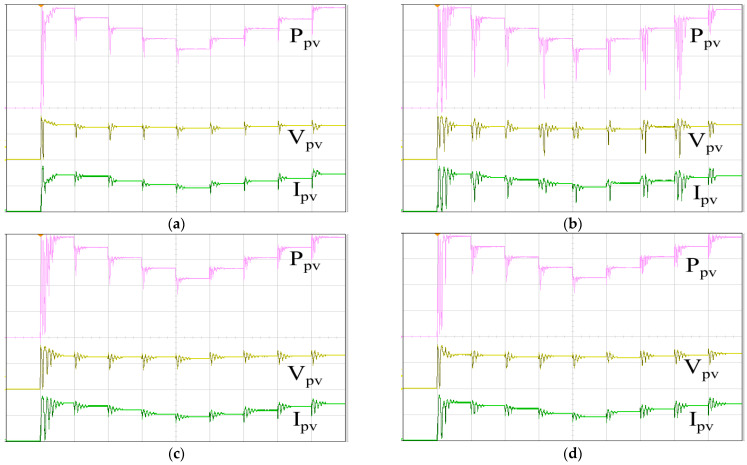
MPPT responses under varying irradiance conditions: (**a**) IQPSO; (**b**) QPSO; (**c**) FA; (**d**) PSO (Ppv: 500 W/div, Vpv: 200 V/div, Ipv: 5 A/div, time: 10 s/div).

Figure 24 displays the MPPT responses under varying irradiance conditions using various algorithms. Figure 24a depicts the response of the IQPSO MPPT algorithm, distinguished by the fastest response and highest accuracy. Figure 24b illustrates the response of the QPSO MPPT algorithm, which exhibits slower response times and lower accuracy. Figure 24c shows the response of the FA MPPT algorithm, known for its speed but lower accuracy. Figure 24d presents the response of the PSO MPPT algorithm, which demonstrates the poorest accuracy and slower response times. Table 7 provides a summary of the experimental results under changing irradiance conditions for comparison. It is evident that when the irradiance conditions fluctuate, the IQPSO proposed in this study consistently demonstrates superior MPPT performance, either with respect to tracking accuracy or tracking time. 

**Figure 25:**
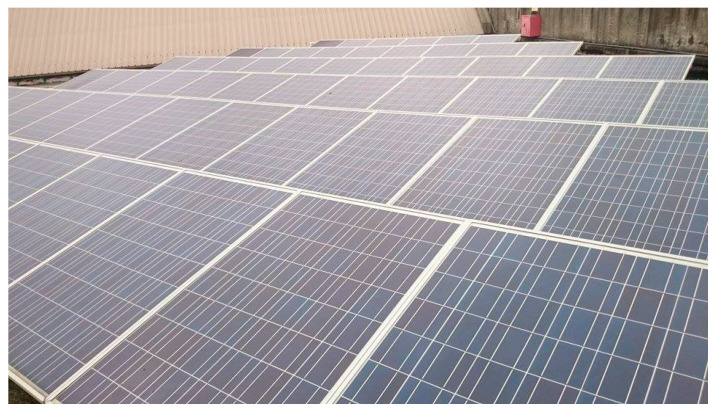
The 2 kW PV arrays.

**Figure 26:**
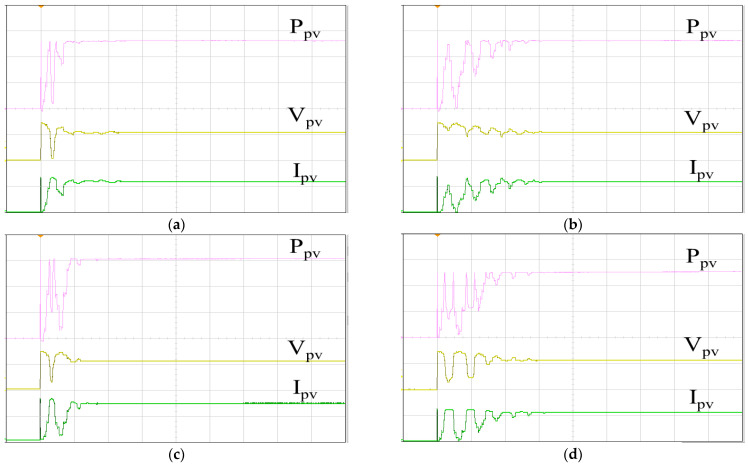
MPPT responses of the PV arrays under single-peak conditions: (**a**) IQPSO; (**b**) QPSO; (**c**) FA; (**d**) PSO (Ppv: 500 W/div, Vpv: 200 V/div, Ipv: 5 A/div, time: 2 s/div).

Figure 25 showcases the actual PV arrays, comprising ten monocrystalline silicon modules connected in series. At a temperature of 25 °C and an irradiance of 1000 W/m^2^, the maximum power output reaches 2 kW. Figure 26 illustrates the tracking responses of the PV arrays under single-peak conditions using various MPPT algorithms. These experiments were conducted under similar ambient conditions (irradiance and temperature), with no shading on the PV arrays. Figure 26a depicts the MPPT response using IQPSO, characterized by the fastest response and highest accuracy. Figure 26b illustrates the MPPT response using QPSO, which exhibits a slower but accurate response. Figure 26c shows the MPPT response using FA, featuring a fast response speed but lower accuracy. Figure 26d presents the MPPT response using PSO, which exhibits the slowest response and less accuracy. Table 8 provides the experimental data presented in Figure 26. In terms of tracking accuracy, IQPSO performs the best while FA performs the worst. Regarding tracking time, IQPSO is the fastest, and PSO is the slowest.

**Figure 27:**
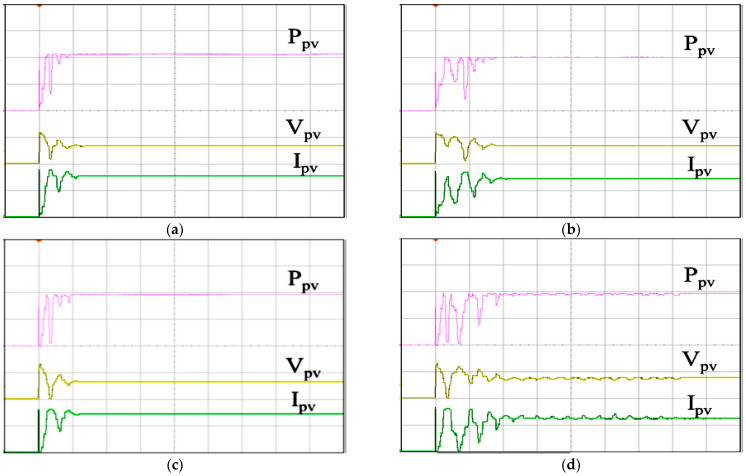
MPPT responses of the PV arrays under multi-peak conditions: (**a**) IQPSO; (**b**) QPSO; (**c**) FA; (**d**) PSO (Ppv: 500 W/div, Vpv: 200 V/div, Ipv: 5 A/div, time: 2 s/div).

Figure 27 shows the tracking responses of the PV arrays utilizing different MPPT algorithms under multi-peak conditions. These experiments were conducted under similar ambient conditions (irradiance and temperature), with the PV arrays partially shaded. Figure 27a illustrates the MPPT response employing IQPSO, exhibiting the quickest response time and the highest level of accuracy. Figure 27b showcases the MPPT response with QPSO, characterized by its accuracy despite a slower response rate. Figure 27c displays the MPPT response with FA, demonstrating a rapid response rate albeit with reduced accuracy. Figure 27d depicts the MPPT response with PSO, exhibiting the poorest tracking accuracy and tracking time. Table 9 provides the experimental data shown in Figure 27. IQPSO demonstrates superior MPPT performance, whereas PSO exhibits the poorest MPPT performance for PV arrays under shading conditions.

**Figure 28:**
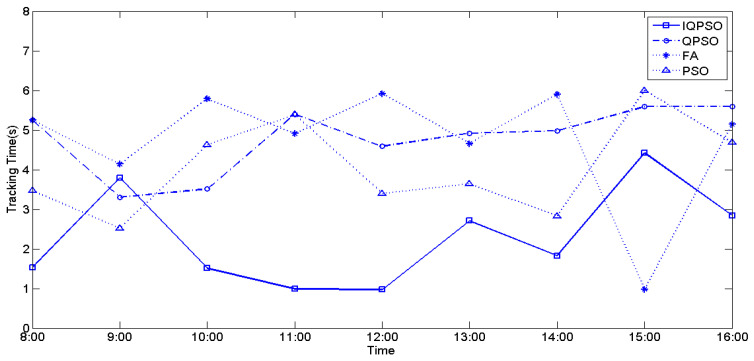
The tracking time on the first day.

**Figure 29:**
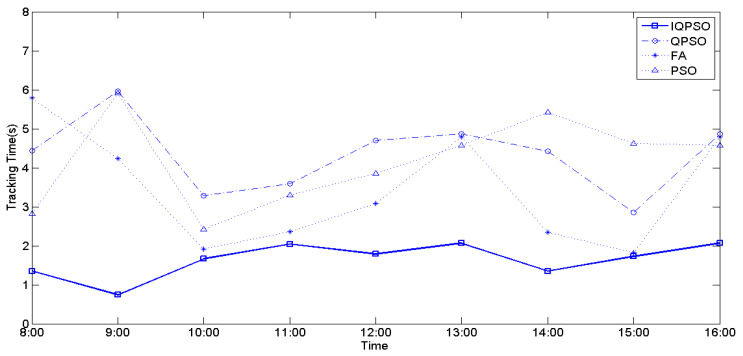
The tracking time on the second day.

**Figure 30:**
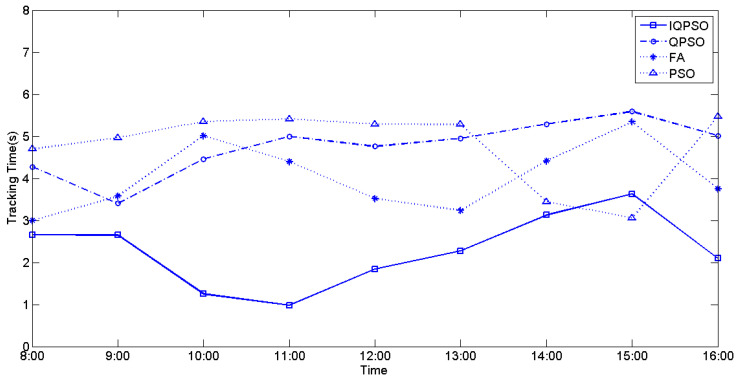
The tracking time on the third day.

The values of irradiance and temperature vary depending on the relative position of the sun and the PV arrays. To compare the MPPT performance using the four biomimetic algorithms under varying irradiance and temperatures, full-day experiments were conducted from 8 a.m. to 4 p.m. MPPT measurements were taken every hour, and the currently measured power represents the maximum generated power of the PV arrays for that hour. Figure 28, Figure 29, and Figure 30 illustrate the tracking time of the full-day MPPT measurements on the first, second, and third days, respectively. Table 10 presents the maximum power generation output of the PV arrays over three days. From these charts, it is evident that utilizing IQPSO for MPPT consistently results in achieving the maximum power generation output and the fastest tracking time each day throughout the three-day period.

## 7. Conclusions

This paper presents the IQPSO algorithm, aimed at enhancing both the tracking accuracy and efficiency of the conventional QPSO approach in the pursuit of optimal solutions. The IQPSO algorithm is applied to a photovoltaic maximum power tracking system, which incorporates a buck-boost converter capable of operating in both buck and boost modes. Therefore, it enables the PGS to operate at the MPP under both single-peak and multi-peak conditions. The investigation compares the maximum power tracking accuracy and tracking time of four bio-inspired optimization methods through single-peak experiments, multi-peak experiments, and irradiance variation experiments. Subsequently, the superiority of the proposed IQPSO in practical PGS applications is validated through single-peak testing, multi-peak testing, and comprehensive full-day testing. Experimental findings conclusively demonstrate that the proposed IQPSO exhibits optimal convergence speed and superior maximum power search capability, thereby significantly enhancing the power generation efficiency of PGS under various conditions such as uniform irradiance, partial shading, and fluctuating irradiance.

To further enhance the power generation of PV panels, future research endeavors may explore the potential impact of employing holographic interferometry to measure the heat transfer from the surfaces of various materials on PV panels to the surrounding air [43]. The algorithm proposed in this paper presents promising prospects for diverse renewable energy applications, with particular relevance to wind energy conversion systems [44]. Confronting variable wind speed conditions requires swift responses to fluctuations in wind speed, thereby presenting a significant challenge. However, the algorithm introduced in this study emerges as a novel solution to address this pressing concern. Furthermore, the proposed algorithm holds potential for future applications in wind turbines utilized for agricultural water pumping [45], which can extract peak power quickly and efficiently under changing wind speeds. Moreover, photovoltaic systems frequently situated in desert climates encounter substantial shading caused by sand and dust accumulation, posing formidable obstacles to power generation [46]. Leveraging its multi-peak processing capabilities, the proposed algorithm demonstrates promising adaptability to enhance the performance of photovoltaic systems in desert environments. Recent years have witnessed a notable surge in interest surrounding the integration of superconducting magnetic energy storage equipment within smart grids [47]. The proposed algorithm’s application spectrum includes mitigating the impact of partial shading on the DC bus side of photovoltaic systems, along with the resolution of load imbalances on the AC bus side. Furthermore, the potential integration of photovoltaic systems with ocean-going vessels represents another promising application realm [48]. Given the prolonged sailing durations and variable environmental factors, including light, temperature, humidity, and climate, the need for swift and precise responses to dynamic shadows becomes paramount. Significantly, the algorithm proposed in this paper is poised to meet this critical demand.

## Figures and Tables

**Table 1:** Specifications of the MPPT circuit.

System Power	2000 W
Input voltage *V_pv_*	10–560 V
Output voltage *V_o_*	100–400 V
Inductor *L_M_*	1.2 mH
Input capacitor *C_in_*	560 μF
Output capacitor *C_out_*	560 μF

**Table 2:** Parameter settings in various MPPT algorithms.

Method	PSO		FA		QPSO		IQPSO	
Parameter	*W*	0.3	*A*	0.02	*β_max_*	1.0	*γ*	2.0
	*C*_1_	0.5	*Β*	0.50	*β_min_*	0.1		
	*C*_2_	0.5	*Γ*	0.50				

**Table 3:** Experimental results in the single-peak case.

	Maximum Power (W)	Tracking Power (W)	Tracking Accuracy	Tracking Time (s)
IQPSO	1999.92	1980.59	99.03 %	1.32
QPSO	1999.92	1969.62	98.48 %	3.93
FA	1999.92	1926.04	96.31 %	2.81
PSO	1999.92	1954.81	97.74 %	4.51

**Table 4:** Ablation study in the single-peak case.

	Tracking Power (W)	Tracking Accuracy	Tracking Time (s)
IQPSO w/o Equation (8) CHG	1970.12	98.51 %	2.91
IQPSO w/o Equation (9) CHG	1977.32	98.87 %	2.32
IQPSO w/o Equation (10) CHG	1972.32	98.62 %	2.51
IQPSO w/o Equation (11) CHG	1976.12	98.81 %	2.38

**Table 5:** Experimental results in the multi-peak case.

	Maximum Power (W)	Tracking Power (W)	Tracking Accuracy	Tracking Time (s)
IQPSO	1260.02	1244.99	98.81 %	1.93
QPSO	1260.02	1239.55	98.38 %	6.78
FA	1260.02	1215.66	96.48 %	2.61
PSO	1260.02	1205.78	95.70 %	5.41

**Table 6:** Ablation study in the multiple-peak case.

	Tracking Power (W)	Tracking Accuracy	Tracking Time (s)
IQPSO w/o Equation (8) CHG	1240.09	98.42 %	4.51
IQPSO w/o Equation (9) CHG	1243.99	98.73 %	2.42
IQPSO w/o Equation (10) CHG	1241.48	98.53 %	3.83
IQPSO w/o Equation (11) CHG	1243.75	98.71 %	3.25

**Table 7:** Experimental results under changing irradiance conditions.

	Average Tracking Accuracy	Average Tracking Time (s)
IQPSO	99.84 %	2.04
QPSO	98.73 %	3.60
FA	98.55 %	2.37
PSO	98.51 %	3.29

**Table 8:** Experimental results of the PV arrays in the single-peak case.

	Irradiance (W/m^2^)	Temperature (°C)	Maximum Power (W)	Tracking Power (W)	Tracking Accuracy	Tracking Time (s)
IQPSO	825	35.0	1314	1307	99.47 %	1.35
QPSO	791	35.8	1317	1304	99.01 %	4.42
FA	780	38.1	1339	1308	98.35 %	2.35
PSO	804	34.5	1272	1255	98.66 %	5.43

**Table 9:** Experimental results of the PV arrays in the multi-peak case.

	Irradiance (W/m^2^)	Temperature (°C)	Maximum Power (W)	Tracking Power (W)	Tracking Accuracy	Tracking Time (s)
IQPSO	986	39.6	1065	1064	99.91 %	1.73
QPSO	1010	37.8	1005	997	99.20 %	3.86
FA	975	38.1	967	948	98.03 %	1.93
PSO	977	38.8	968	941	97.21 %	4.63

**Table 10:** Experimental results for maximum power generation over three days (Wh).

	IQPSO	QPSO	FA	PSO
Day1	6703.66	6388.45	6422.04	6000.40
Day2	10,521.22	10,408.85	10,319.25	10,164.69
Day3	9382.16	9284.92	9211.54	9072.42
Total	26,607.04	26,082.22	25,952.83	25,237.51

## Data Availability

Data are contained within the article.
